# Different routes to liking: how readers arrive at narrative evaluations

**DOI:** 10.1186/s41235-022-00419-0

**Published:** 2022-07-30

**Authors:** Marloes Mak, Myrthe Faber, Roel M. Willems

**Affiliations:** 1 Centre for Language Studies, Radboud University Nijmegen, Erasmusplein 1, 6525 HT Nijmegen, The Netherlands; 2 Donders Institute for Brain, Cognition and Behaviour, Radboud University Nijmegen, Kapittelweg 29, 6525 EN Nijmegen, The Netherlands; 3 Department of Communication and Cognition, Tilburg Center for Cognition and Communication, Tilburg University, Warandelaan 2, 5037 AB Tilburg, The Netherlands; 4 Max Planck Institute for Psycholinguistics, Wundtlaan 1, 6525 XD Nijmegen, The Netherlands

**Keywords:** Appreciation, Narratives, Reading, Bayesian multilevel modeling, Literature

## Abstract

When two people read the same story, they might both end up liking it very much. However, this does not necessarily mean that their reasons for liking it were identical. We therefore ask what factors contribute to “liking” a story, and—most importantly—how people vary in this respect. We found that readers like stories because they find them interesting, amusing, suspenseful and/or beautiful. However, the degree to which these components of appreciation were related to how much readers liked stories differed between individuals. Interestingly, the individual slopes of the relationships between many of the components and liking were (positively or negatively) correlated. This indicated, for instance, that individuals displaying a relatively strong relationship between interest and liking, generally display a relatively weak relationship between sadness and liking. The individual differences in the strengths of the relationships between the components and liking were not related to individual differences in expertize, a characteristic strongly associated with aesthetic appreciation of visual art. Our work illustrates that it is important to take into consideration the fact that individuals differ in how they arrive at their evaluation of literary stories, and that it is possible to quantify these differences in empirical experiments. Our work suggests that future research should be careful about “overfitting” theories of aesthetic appreciation to an “idealized reader,” but rather take into consideration variations across individuals in the reason for liking a particular story.

**Supplementary Information:**

The online version contains supplementary material available at 10.1186/s41235-022-00419-0.

## Significance statement

When picking a book to read, people often rely on the recommendation of others, either in person or through online reviews. However, two people reading the same book might (dis)like it for entirely different reasons. Questions used to capture these evaluations, such as whether one “likes” a story, do not tap into these different routes to appreciation. In our work, we empirically quantify the individual differences in routes to “liking.” We found that readers indeed differ in their reasons for liking a story. This variation can be taken as a starting point for future work into how people come to like or dislike certain books or narratives.

## Introduction

People often do not have to think long about whether they like something (e.g., architecture, art; see A. Jacobs et al., 2016). Indeed, it seems easy for readers to decide whether they “like” a story or not. Although such ratings of liking can give us an impression of someone’s aesthetic preferences, they do not offer any insight into what drives these evaluations. People might arrive at the same judgment in different ways: it is possible that someone for instance likes a story because of its emotional content, whereas another person likes it because they are interested in the topic. Here, we aim to explore how people differ in what determines whether they “like” a story or not in the context of literary reading. We particularly investigate whether and how the contribution of different cognitive and emotional processes varies across readers.

Models of aesthetic appreciation propose that both cognitive and affective processes play a role in aesthetic evaluation (Chatterjee & Vartanian, 2016; Jacobs, 2015a; Leder & Nadal, 2014; Leder et al., 2004), and that both of these processes can be either conscious or subconscious (i.e., automatic; see also Graf & Landwehr, 2015). In addition, sensory-motor processes, such as sensation and perception, might play a role, in particular in the context of engaging with aesthetic objects such as artworks (Chatterjee & Vartanian, 2016). How these processes interact with each other likely varies across individuals. For instance, expertize, taste, personality, and pre-existing mood are likely to affect how cognitive and affective processes influence evaluative decisions made by observers (Chatterjee & Vartanian, 2016; Leder et al., 2004). An art connoisseur for instance will experience a painting differently than a layman (and arrive at their evaluative decision differently): the connoisseur may rely more heavily on cognitive processes (e.g., trying to understand the meaning of the painting) whereas the layman may rely more on the positive or negative affect elicited by the painting (see Leder et al., 2014, for evidence of reduced affective responses to artworks in art history students). This means that their aesthetic experience of the painting might differ, *even* if they both reach the same conclusion about the painting (“I like this painting”).

Cognitive and affective processes are also thought to play a role in how people arrive at aesthetic evaluations in narrative reading (Jacobs, 2015a). According to the Neurocognitive Poetics Model (NCPM; Jacobs, 2015b), the fast, affective processing route results in “fiction feelings” (e.g., empathy, vicarious emotions, narrative absorption) via emotional contexts in narratives. Cognitive processing is thought to be a slow route that results in so-called aesthetic feelings (i.e., feelings induced by the aesthetic experience) via foregrounded elements in narratives (i.e., stylistic devices, defamiliarization). Previous work has suggested that motivational-emotional processes such as interest, meta-emotions, and taste might influence whether people are likely to prefer reading narratives that align with either route (Bartsch et al., 2008; Zillmann, 1988), but empirical evidence is markedly lacking in the field (Jacobs, 2015b).

Recent work has approached aesthetic emotions as a multidimensional construct, resulting in the development of the Aesthetic Emotions Scale (AESTHEMOS; Schindler et al., 2017). This scale captures emotions related to aesthetics (e.g., positive emotions such as beauty, fascination, and negative emotions such as ugliness), epistemics (e.g., interest), amusement (e.g., humor), and qualitative aspects of experience such as whether the reader feels activated or relaxed by the text (Schindler et al., 2017). Importantly, experiencing one emotion does not preclude the possibility of experiencing another (seemingly opposite) emotion (Schindler et al., 2017). Applications in the context of various aesthetic experiences (e.g., concerts, theatrical performances, exhibitions) highlight how people can experience many different emotions at the same time, and that the specific combination of experienced emotions can differ between people and between (types of) stimuli, together constituting a person’s “signature” of affective aesthetic processing (Schindler et al., 2017).

Here, we aim to explore how people differ in what determines whether they “like” a story or not. We build on previous work that measured “aesthetics from below” (Knoop et al., 2016; cf. Fechner, 1876). Knoop and colleagues (2016) selected adjectives that could be used to describe readers’ aesthetic experiences while engaging with literature (i.e., poetry, plays, comedies, novels, short stories). Ratings were gathered from 1544 participants, resulting in a list of 22 adjectives that were brought up by a minimum percentage (> 10%) of participants (Knoop et al., 2016; for a similar approach to capture the aesthetic appreciation of objects, see Jacobsen et al., 2004). From these lists, we took all adjectives (*N* = 13) that could be used for rating literary short stories (thus leaving out musical/poetry specific terms such as *melodious* or *poetic*^1^) and presented them, together with a question regarding general story liking, to 270 readers who read Dutch literary short stories (nine different stories in total) across three experimental studies. Since it is unclear how readers differ in their reliance on one or more aesthetic features to come to an overall ‘liking’ of a story, the main goal of our paper will be to get better insight into such individual differences.

In this paper, we aim to answer five consecutive questions, to uncover what aspects of stories lead to story liking, and, importantly, whether and how this differs between readers. We ask (1) whether the adjectives derived from Knoop and colleagues (2016) tap into distinguishable components of literature appreciation. We obtain these components using principal components analysis, which results in clusters of adjectives and participant-level scores on each component. We ask (2) how these components are related to “story liking,” and (3) whether there is variation between readers in how the components relate to story liking. Subsequently, we ask whether (4) the direction of the relationship between the components and liking is consistent across participants and (5) whether the variation in slopes between participants is systematically associated with reader characteristics (i.e., reading habits, print exposure, story world absorption). This last question sheds light on whether literary expertize matters with regard to how different components of appreciation contribute to the aesthetic evaluation of stories.

## Methods

Datasets collected in three previous studies were combined for this investigation. In all the previous studies participants read Dutch literary short stories, and completed an appreciation questionnaire (Mak & Willems, 2019) as well as questionnaires regarding story world absorption (Kuijpers et al., 2014), reading habits in daily life (Hartung et al., 2016; Mak & Willems, 2019), and print exposure (Author Recognition Test; Stanovich & West, 1989). We will describe each questionnaire in more detail below.

The first study (Mak & Willems, 2019) investigated different kinds of mental simulation during narrative reading, the second study (Eekhof et al., 2018) tested the influence of verb tense on mental simulation during literary reading, and the third study (Mak et al., 2020) investigated the influence of prereading instructions on reported mental imagery and other subjective reading experiences.

### Participants

In total, 270 native speakers of Dutch were tested across three experimental studies (see Table 1 for sample characteristics). The majority of the participants were university or college students. Depending on the study, participants read two, three or four Dutch literary short stories (resulting in a total of nine different stories overall, for distribution across studies, see below), which resulted in 716 individual data points (i.e., completed questionnaires; one per participant/story combination). Of these 716 questionnaires, there were 13 questionnaires where at least one question was skipped by the participant. As a result, 703 data points were complete and could be entered into data analysis. Participants were recruited from the Radboud University participant pool, and received appropriate compensation (monetary or course credits) for their participation. All studies were approved by the local ethics committees (approval code 8976) and were conducted in accordance with the Declaration of Helsinki.

**Table 1:** Sample information for the three studies

Study	*N*			*M*_age_ (range)
	Female	Male	Other	
Study 1	81	21	0	23.27 (18 – 40)
(Mak & Willems, 2019)				
Study 2	33	9	1	23.26 (18 – 46)
(Eekhof et al., 2018)				
Study 3	103	22	0	23.80 (18 – 61)
(Mak et al., 2020)				

### Materials

#### Stories

Characteristics of the stories read by the participants in the three studies are shown in Table 2. A short synopsis of all stories can be found in Additional file 1: Synopsis. The common structure of all stories is that they describe an event or person, followed by some plot twist or extraordinary event, and end with a very open ending that leaves the reader feeling a bit alienated. Stories differed across studies, as they had been selected separately for each study, from the entire collection of Dutch literary short stories. However, all studies used literary stories, written by critically acclaimed authors and published by literary publishing houses. All stories belonged to the genre of “literary short story,” were available in Dutch, and were readable in 10 to 15 min. Except for *Symbols and Signs,* all stories were originally written in Dutch. *Symbols and Signs* was read in a published translation, which was translated from English to Dutch by a professional translator. In Study 1 and Study 3, the stories were presented in their original form. In Study 2, the original stories, alongside slightly altered versions in which the verb tense was changed from present to past tense or vice versa (for reasons not relevant to the current study, and with no reported difference in readability between original and altered versions, see Eekhof et al., 2018).

**Table 2:** Descriptive information for the stimulus stories used in the three previous studies

Study	Story	Author	Year of publication	Word count
Study 1 (Mak & Willems, 2019)	De mensen die alles lieten bezorgen (The people that had everything delivered)	Rob van Essen (2014)	2014	2988
	De Chinese bruiloft (The Chinese wedding)	Sanneke van Hassel (2012)	2012	2659
	Signalen en symbolen (Symbols and signs)	Vladimir Nabokov 2003)	1948/2003	2143
Study 2 (Eekhof et al., 2018)	Het is muis (It is mouse)	Sanneke van Hassel (2012)	2012	2016
	Hoe de wolven dansen (How the wolves dance)	Jordi Lammers (2017)	2017	1176
	De invaller (The substitute)	René Appel (2003)	2003	743
	Ze is overal (She is everywhere)	Ed van Eeden (2015)	2015	1074
Study 3 (Mak et al., 2020)	Brommer op zee (Moped on sea)	Maarten Biesheuvel (1972)	1972	1827
	God en de gekkenrechter (God and the judge of the insane)	Adriaan van Dis (1986)	1986	2026

#### Questionnaires

The Appreciation Questionnaire consisted of a general score of story liking (*How did you like the story*; 1 = It was very bad, 7 = It was very good) and 13 adjectives (e.g., *[did you find the story] Entertaining,… Ominous, *etc*.*) that we adapted from Knoop and colleagues (2016). Studies 2 (Eekhof et al., 2018) and 3 (Mak et al., 2020) both omitted one adjective from the list (Study 2: *Special*; Study 3: *Entertaining*), resulting in 11 adjectives that were included in the lists in all three studies. The resulting 11 adjectives that were included in the analysis can be found in Table 3. Finally, six questions were asked regarding the enjoyment of the story (from Kuijpers et al., 2014; e.g., *I was constantly curious about how the story would end; I thought the story was written well, *etc*.*). These final six questions were omitted from the analyses in the current study, because they were highly correlated with the *liking* question, and were therefore not considered to be of added importance for the current investigation. Participants rated both the adjectives and the questions regarding enjoyment on a seven-point scale (1 = disagree, 7 = agree).

**Table 3:** Pattern matrix for the PCA of the 11 adjectives on the appreciation questionnaire (*N* = 703)

	Pattern matrix				
	Interest	Sadness	Suspense	Amusement	Beauty
Beautiful	0.11	− 0.01	− 0.12	0.02	**0.92**
Boring	**− 0.90**	− 0.01	− 0.01	0.01	− 0.06
Deeply moving	0.20	0.35	**0.41**	− 0.03	0.32
Funny	0.25	− 0.01	− 0.25	**0.84**	− 0.17
Interesting	**0.51**	0.05	0.18	0.13	0.32
Ominous	− 0.05	0.12	**0.88**	− 0.05	− 0.10
Sad	− 0.03	**0.93**	− 0.09	− 0.03	0.04
Suspenseful	0.29	− 0.12	**0.75**	0.08	− 0.02
Tragic	0.04	**0.91**	0.08	0.04	− 0.07
Witty	− 0.18	0.00	0.24	**0.83**	0.25
Captivating	**0.59**	0.00	0.25	0.16	0.24

Factor loadings over .40 appear in bold

To compare the results on the appreciation questionnaire to other subjective reading experiences, we also measured story world absorption, which refers to an experiential state in which readers are focused on reading and the content of what is read (Kuijpers, 2014). In particular, if the reading process feels effortless, readers experience a narrative world and feel for or with characters, and mental imagery is rich and vivid (Kuijpers, 2014). Story world absorption was measured using the Story World Absorption Scale (SWAS; Kuijpers et al., 2014). The SWAS is a validated scale consisting of 18 items with high internal validity, which measure four aspects of story world absorption on the four subscales Attention, Transportation, Emotional Engagement and Mental Imagery (e.g*., When I finished the story I was surprised to see that time had gone by so fast; I could imagine what the world in which the story took place looked like*). Participants rated each question on a seven-point scale (1 = disagree, 7 = agree).

Additionally, we were interested in whether habitual readers differed in their appreciation of stories from participants who do not read much in daily life. Reading habits were measured using five multiple choice questions about reading habits in everyday life, with four or five answer options (Hartung et al., 2016; Mak & Willems, 2019; e.g., *How often do you read fiction?; How many books do you read each year?*). Additionally, participants were asked for their genre preference in an open-ended question, where they could list up to three genres they enjoyed reading (this question was added for purposes irrelevant to the current study, and will not be used in the analyses in this paper). As an implicit measure of print exposure, participants completed the well-established Author Recognition Test (ART; Stanovich & West, 1989; Acheson et al., 2008; Dutch adaptation reported in Koopman, 2015), consisting of 42 names (30 real authors and 12 foils), where they had to indicate who they thought were genuine authors.

### Procedure

In all studies, informed consent was obtained before the experiment, after which participants were instructed to read as naturally as possible. The stories (i.e., three stories in Study 1, four stories in Study 2 and two stories in Study 3; see Table 2) were read in a counterbalanced order. After reading the first story, participants completed the SWAS and Appreciation Questionnaire. These steps were repeated for the other stories in the experiment. After participants had read the last story and completed the corresponding questionnaires, they filled out the reading habits questionnaire and the Author Recognition Test.

### Data Analysis

**Fig. 1:**
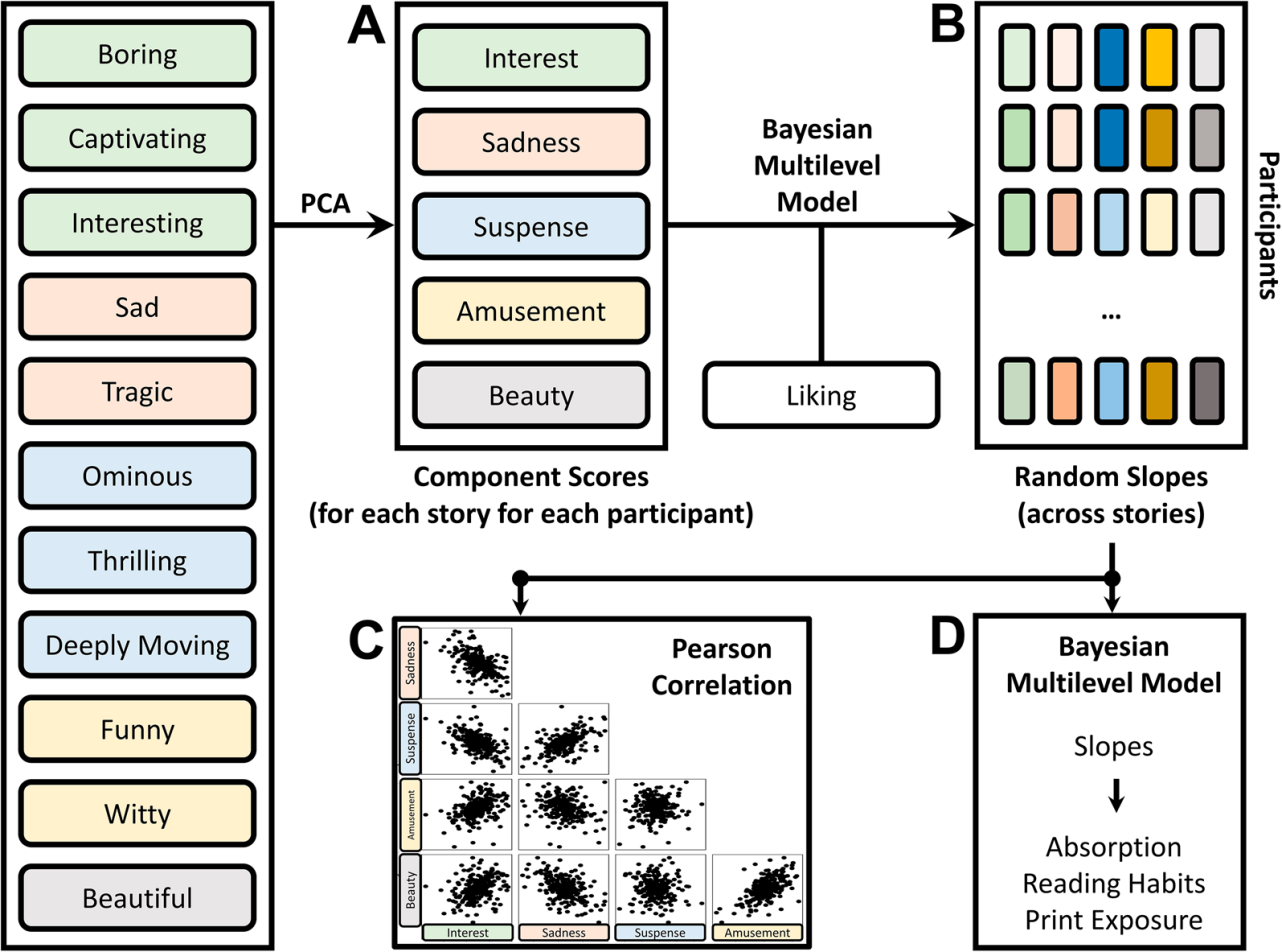
Visualization of the Analysis Pipeline. *Note* The arrows indicate the order of processing steps

In Fig. 1, we give a schematic overview of the analysis pipeline. Each analysis step is described in detail below, the following description serves to give a rough overview. In the first step of the analysis, the 11 adjectives from the appreciation questionnaire (see Fig. 1, left column) were entered into a principal components analysis resulting in five components (Fig. 1A). Then, participants’ scores (per story) on these components were linked to liking scores per story, while allowing for random slopes for the components over participants and over stories (Fig. 1B). With this analysis, we first focused on the population-level effects of the components, to find out whether the different components of appreciation each play a role in the eventual evaluation of stories. The by-participant variation in the random slopes across components was compared in a correlation analysis (Fig. 1C). Finally, variation in the random slopes was linked to absorption, reading habits and print exposure (Fig. 1D). With this final analysis, we zoom in on the participant level to acknowledge the individual differences in story liking and to try to explain some of these individual differences by linking them to concepts that are theorized to be related to aesthetic processes and may explain individual differences therein.

## Results

All data and analysis scripts are available on the Open Science Framework, https://osf.io/h3ct6/.

### Question 1: Do adjectives tap into distinguishable components of literature appreciation?

The first step of the analysis pipeline (see Fig. 1) was to reduce the 11 adjectives to a smaller number of components consisting of highly similar adjectives (Fig. 1A). Using the package *psych* (Revelle, 2020) in *R* version 4.0.3 (R Core Team, 2021), we conducted a principal component analysis (PCA) with oblique rotation (direct oblimin) on the 11 appreciation adjectives used in all three studies. The resulting components tap into distinct aspects of literature appreciation.

The Kaiser–Meyer–Olkin measure (KMO) was 0.83 (all KMO values for individual items > 0.61), indicating good sampling adequacy for this analysis. Bartlett’s test of sphericity showed sufficient correlation between items, χ2 (55) = 490.56, *p* < 0.001. The primary rationale for determining the number of components was maximization of explained variance (at least 80% of variance explained), along with interpretability of the component (i.e., reducing the number of dimensions while making sure that these components still represented the original data reasonably well). A 5-component solution explained 81% of the variance and therefore represents the original data closely. For the 5-component solution, the mean communality was > 0.7, and the fit (fit based upon off diagonal values) was 97.2%.

The first component that we found corresponded to interest (consisting of items boring (-), captivating, and interesting); the second component to sadness (Sad, Tragic); the third component to suspense (ominous, suspenseful, deeply moving); the fourth component to amusement (Funny, Witty); and the final component to beauty (Beautiful). The structure and pattern matrices for the factor loadings after rotation can be found in Table 3. All correlations between the components were below *r* = 0.43, confirming that the extracted components were indeed measuring separate constructs, and that such lists of adjectives can be used to measure distinct aspects of literature appreciation. Component scores per participant per story were used in the subsequent analyses.

### Question 2 and 3: How do adjective components relate to “story liking”? Is there variation between readers in the way these components relate to “story liking”?

The components resulting from the PCA were used to assess how the adjectives related to “story liking.” This relationship was analyzed (see Fig. 1B) with a Bayesian Multilevel^2^ Model using the package *brms* (Bürkner, 2017, 2018) and *Stan* (Stan Development Team, 2020) in *R* version 4.0.3 (R Core Team, 2021). The rationale for calculating a Bayesian multilevel model as opposed to a “classical” frequentist model was that Bayesian models are more flexible and more capable of fitting complex models (e.g., Bürkner, 2018; Nalborczyk et al., 2019). Rather intuitively, Bayesian multilevel models calculate the range of the most probable values of each parameter, a 95% Credible Interval. If this Credible Interval does not cross zero for a given parameter, this indicates a 95% certainty that the true value of this parameter is distinguishable from zero.

We constructed a partially crossed model that predicted the answer on the general liking question (*How did you like the story?*) by the individual scores on the five components found in step 1, allowing random intercepts and slopes for all five predictors per participant and per story.^3^ This random effect structure made sure that the model took the between subject and between story variation into account. As a result, the data were analyzed in such a way that the observations that belonged together (because they belonged to the same participant) were grouped together. Therefore, these random intercepts allowed us to control for the fact that all participants and all stories occurred more than once in the dataset. For the population-level intercept, we used a weakly informative, normally-distributed prior with a mean of 0 and a standard deviation of 10. A weakly informative, normally-distributed prior with a mean of 0 and a standard deviation of 1 was set for the fixed effects. These priors are considered relatively conservative (McElreath, 2016). As variance can only be positive, weakly regularizing, half-cauchy priors with a mean of 0 and a standard deviation of 1 were used for the variance of the random effects as well as the overall variance (as suggested by Gelman, 2006; McElreath, 2016). The model was trained during 4000 iterations, using 4 chains, and using an MCMC sampler (for a complete model specification, see the analysis scripts on the Open Science Framework, https://osf.io/h3ct6/). The Gelman-Rubin diagnostic (*Rhat*) was 1.0 for all parameters, indicating that the model had converged.

**Fig. 2:**
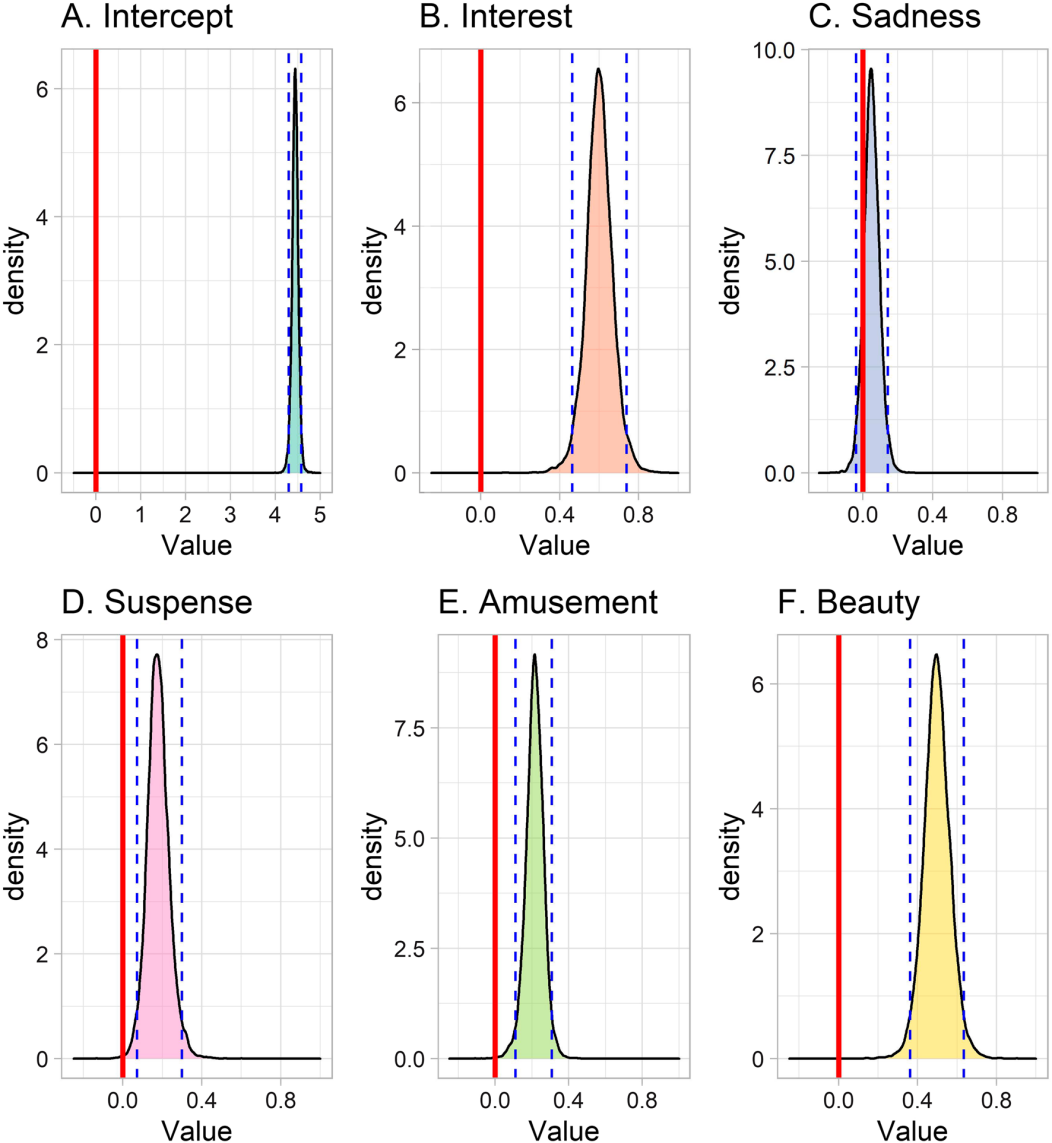
Posterior distributions of the population-level fixed effects of the relationships between the components and liking. *Note* The Intercept (A) represents the average liking score. The blue dashed lines indicate the limits of the 95% credible interval. If the credible interval of a parameter does not cross zero, this means that it is likely that the true value for that parameter is different from zero. Code for this figure is adapted from https://www.rensvandeschoot.com/tutorials/brms-started/

We found that the interest component was positively associated with story liking, showing that stories that were considered more interesting were generally liked more (Table 4; Fig. 2B; mass > 0: 99.9%). Additionally, the relationship between interest and liking varied between participants and between stories (the standard deviation of the slope of the interest component = 0.16 [CI 0.02–0.28] across participants; and 0.15 [CI 0.03–0.32] across stories).^4^ Posterior distributions of the individual slopes for the association between interest and liking (per participant) showed that this association was positive for all participants (all participants showed a positive association between interest and general story liking; the complete by-participant posterior distributions can be seen in Additional file 2: Fig. S1).

**Table 4:** Posterior distributions (Median, MAD, 95% CI) of the population-level associations between the components and liking

	Estimate (Median)	Estimate (MAD)	Lower bound (95%CI)	Upper bound (95%CI)
(Intercept)	4.44	0.06	4.30	4.58
Interest	0.60	0.06	0.47	0.73
Sadness	0.05	0.04	− 0.03	0.14
Suspense	0.17	0.05	0.08	0.30
Amusement	0.22	0.05	0.11	0.31
Beauty	0.50	0.06	0.36	0.63

*MAD* Median absolute deviation; *CI* Credible interval

We found no conclusive evidence for an association between the sadness component and story liking (Table 4; Fig. 2C): as the credible interval crossed zero, we cannot reasonably assume a positive relationship between sadness and liking (mass > 0: 87.9%). However, we did find variation between participants (the standard deviation of the slope of the sadness component = 0.23 [CI 0.11–0.32] across participants). The posterior distributions of the individual slopes for the association between sadness and liking (per participant) showed that some participants showed a positive association between sadness and liking, although there were also participants who showed no association or a negative association between sadness and liking (the complete by-participant posterior distributions can be seen in Additional file 3: Fig. S2). Ultimately, the data suggest that some readers like a story more when they consider it to be sadder, whereas others are indifferent to the sadness of a story or actually dislike sad stories. There was no clear variation in the relationship between sadness and liking across stories (the standard deviation of the slope of the sadness component = 0.05 [CI 0.00–0.17] across stories).

The suspense component was positively associated with story liking (see Table 4; Fig. 2D; mass > 0: 99.7%). The relationship between suspense and liking varied between participants and between stories (the standard deviation of the slope of the suspense component = 0.18 [CI 0.04–0.28] across participants; and 0.09 [CI 0.01–0.27] across stories). The posterior distributions of the individual slopes for the association between suspense and liking (per participant) suggested that a large part of the participants showed a positive association between suspense and liking, but there were also participants who showed no association or a negative association between suspense and liking (the complete by-participant posterior distributions can be seen in Additional file 4: Fig. S3). This suggests that many readers like a story more when they consider it to be more suspenseful, but some are indifferent to suspense, or dislike suspenseful stories.

The amusement component showed a very similar pattern. Amusement was positively associated with story liking (see Table 4; Fig. 2E; mass > 0: 99.8%). Again, the relationship between amusement and liking varied between participants (the standard deviation of the slope of the amusement component = 0.20 [CI 0.08–0.29] across participants). The posterior distributions of the individual slopes for the association between amusement and liking suggested that a large part of the participants showed a positive association between amusement and liking, whereas some participants showed no association or a negative association between amusement and liking, indicating that many readers like a story more when they consider it to be more amusing, but some are indifferent, or dislike amusing stories (the complete by-participant posterior distributions can be seen in Additional file 5: Fig. S4). There was no clear variation in the relationship between amusement and liking across stories (the standard deviation of the slope of the amusement component = 0.07 [CI 0.00–0.22] across stories).

Finally, the beauty component was positively associated with story liking, showing that stories that were considered more beautiful were generally liked more (see Table 4; Fig. 2F; mass > 0: 99.98%). The relationship between beauty and liking varied between participants and between stories (the standard deviation of the slope of the beauty component = 0.16 [CI 0.03–0.27] across participants; and 0.14 [CI 0.06–0.30] across stories). The posterior distributions of the individual slopes for the association between beauty and liking (per participant) showed that this association was positive for all participants (all participants showed a positive association between beauty and general story liking; the complete by-participant posterior distributions can be seen in Additional file 6: Fig. S5).

### Question 4: Is the direction of the relationship between the components and liking consistent across participants?

**Fig. 3:**
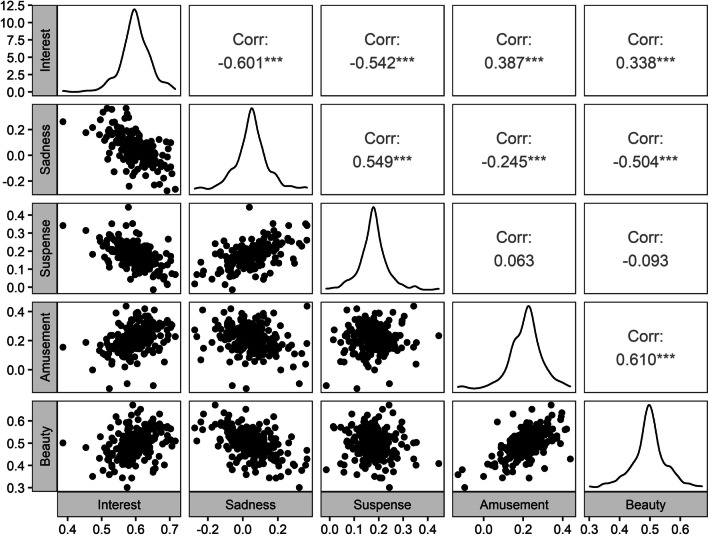
Plot of the correlations between the slopes for the associations of the components and liking. *Note* Below the diagonal, scatterplots of the individual slopes are displayed. The diagonal represents density plots of the distributions of the slopes. Pearson correlation coefficients are given above the diagonal. *** indicates *p* < .001. Bonferroni correction for multiple comparisons was applied

As the relationships between all components and liking reliably varied between participants, it would be interesting to know whether these relationships correlated with each other on the individual level (within participants). For instance, if a given participant displays a relatively strong association between interest and liking, does this same participant also display a relatively strong association between amusement and liking? To address this question, we first extracted the estimated slopes (median per participant, collapsed across the individual story-readings within each participant) for the associations between the components and general story liking (i.e., 270 coefficients for each of the five components) from the model reported above. All slopes were entered into a pair-wise correlation analysis (see Fig. 1C; Fig. 3), with Bonferroni correction for multiple comparisons.

The slopes for the relationship between interest and liking were moderately negatively correlated to the slopes for the relationships between sadness and liking (*r* = − 0.601; *p* < 0.001) and suspense and liking, (*r* = − 0.542; *p* < 0.001), suggesting that participants displaying a relatively strong association between interest and liking, displayed relatively weak associations between sadness / suspense and liking. Oppositely, the slopes for the relationship between interest and liking were weakly positively correlated to the slopes for the relationships between amusement and liking (*r* = 0.387; *p* < 0.001) and beauty and liking (*r* = 0.338; *p* < 0.001), suggesting that participants displaying a relatively strong association between interest and liking, also displayed relatively strong associations between amusement / beauty and liking.

The slopes for the relationship between sadness and liking were moderately positively correlated to the slopes for the relationship between suspense and liking (*r* = 0.549; *p* < 0.001), suggesting that relatively high associations between sadness and liking co-occurred with relatively high associations between suspense and liking. Oppositely, the slopes for the relationship between sadness and liking were weakly negatively correlated to the slopes for the relationship between amusement and liking (*r* = − 0.245; *p* < 0.001) and moderately negatively correlated to the slopes for the relationship between beauty and liking (*r* = − 0.504; *p* < 0.001), suggesting that participants displaying a relatively strong association between sadness and liking, displayed relatively weak associations between amusement / beauty and liking.

The slopes for the relationship between suspense and liking were not correlated to the slopes for the relationships between amusement and liking (*r* = 0.063; *p* = 0.30) and between beauty and liking (*r* = − 0.093; *p* = 0.13). This indicates that participants displaying a relatively strong association between suspense and liking, do not reliably display weaker or stronger associations between amusement / beauty and liking.

Finally, the slopes for the relationship between amusement and liking were moderately positively correlated to the slopes for the relationship between beauty and liking (*r* = 0.610; *p* < 0.001). Relatively high associations between amusement and liking co-occurred with relatively high associations between beauty and liking.

### Question 5: Is variation in slopes between participants systematically associated with reader characteristics?

To assess whether the variation in the slopes (between participants) was systematically associated with reader characteristics, we linked the median estimated slopes per participant (see Question 4) to the scores per participant for story world absorption (*M* = 4.25, *SD* = 1.07, range 1.22–6.72), print exposure (Author Recognition Test; *M* = 7.40, *SD* = 4.42, range 0–23), and reading habits (the scores on the Reading Habits questionnaire were z-transformed, as they were measured on slightly varying scales across experiments.

We compared the individual slopes to the reader characteristics with Bayesian Multilevel Models (see Fig. 1D) using the package *brms* (Bürkner, 2017, 2018) and *Stan* (Stan Development Team, 2020). We constructed multilevel models that predicted average scores for story world absorption, reading habits and print exposure by the median estimated slopes per participant for the associations between all five components and liking. For story world absorption, there was more than one observation per participant and per story. Therefore, random intercepts for Participant and Story were included in the model for story world absorption. We used a weak, normally-distributed prior with a mean of 0 and a standard deviation of 10 for the population-level intercept. A normal prior with a mean of 0 and a standard deviation of 1 was set for the fixed effects. These priors are considered relatively conservative (McElreath, 2016). As variance can only be positive, half-cauchy priors with a mean of 0 and a standard deviation of 1 were used for the overall variance (as suggested by Gelman, 2006; McElreath, 2016), as well as the variance of the random effects (in the model for story world absorption). The model was trained during 4000 iterations, using 4 chains, and using an MCMC sampler (for a complete model specification, see the analysis scripts on the Open Science Framework, https://osf.io/h3ct6/). The Gelman-Rubin diagnostic (*Rhat*) was 1.0 for all parameters, indicating that the model had converged.

The variation in the slopes for the relationships between the components and liking were not reliably associated with story world absorption (see Table 5),^5^ print exposure (see Table 6), or reading habits (see Table 7), all credible intervals crossed zero (see Tables 5, 6 and 7 for the mass > 0 for all posterior distributions). This means that the by-participant variability in slopes for the relationships between the components and liking cannot be explained by the variability in the measured reader characteristics.

**Table 5:** Posterior distributions of the associations between the slopes and absorption

	Estimate (Median)	Estimate (MAD)	Lower bound (95%CI)	Upper bound (95%CI)	Mass > 0 (%)
(Intercept)	4.54	0.64	3.28	5.81	99.9
Interest Slope	0.16	0.82	− 1.41	1.75	58.0
Sadness Slope	− 0.45	0.52	− 1.47	0.56	18.4
Suspense Slope	0.55	0.71	− 0.84	1.90	78.5
Amusement Slope	0.02	0.54	− 1.09	1.09	51.4
Beauty Slope	− 0.99	0.70	− 2.44	0.51	9.2

The median, median absolute difference, 95%CI and mass > 0 of the posterior distribution are given

**Table 6:** Posterior distributions of the associations between the slopes and print exposure

	Estimate (Median)	Estimate (MAD)	Lower bound (95%CI)	Upper bound (95%CI)	Mass > 0 (%)
(Intercept)	7.11	0.83	5.44	8.81	99.9
Interest Slope	0.03	0.96	− 1.88	1.94	51.3
Sadness Slope	0.15	0.94	− 1.68	2.02	56.5
Suspense Slope	0.00	0.98	− 1.92	1.98	50.3
Amusement Slope	0.53	0.96	− 1.29	2.47	71.2
Beauty Slope	0.26	0.97	− 1.70	2.18	61.3

The median, median absolute difference, 95%CI and mass > 0 of the posterior distribution are given

**Table 7:** Posterior distributions of the associations between the slopes and reading habits

	Estimate (Median)	Estimate (MAD)	Lower bound (95%CI)	Upper bound (95%CI)	Mass > 0 (%)
(Intercept)	− 0.26	0.60	− 1.46	0.93	33.4
Interest Slope	0.20	0.79	− 1.35	1.73	59.0
Sadness Slope	− 0.21	0.49	− 1.19	0.78	33.4
Suspense Slope	− 0.15	0.68	− 1.20	1.48	57.9
Amusement Slope	0.32	0.54	− 0.79	1.42	71.9
Beauty Slope	0.13	0.75	− 1.29	1.59	58.0

The median, median absolute difference, 95%CI and mass > 0 of the posterior distribution are given

## Discussion

In this study, we aimed to determine what makes readers consider a story to be good or bad, and how people differ in this respect. We found that adjectives used in previous studies (e.g., Knoop et al., 2016) tapped into distinguishable components of literature appreciation, that we labeled interest, sadness, suspense, amusement and beauty. Four out of five of these components (i.e., interest, suspense, amusement, beauty) were significantly positively associated with the general question regarding how much participants liked the story. However, interest and beauty were more strongly associated with story liking than the other components (i.e., suspense and amusement). Additionally, although sadness was on average not associated with liking, here we found individual variation as well, with some participants showing a positive association between sadness and liking, and some participants a negative association. When looking at individual slopes per participant, we discovered substantial variation in the associations between the five components and story liking on the individual level, suggesting that there might be distinct patterns of relative associations between these components and story liking.

### Individual Differences in the Routes to Appreciation

We found that the individual slopes between the components on the one hand, and liking on the other hand, were weakly to moderately correlated. For some sets of components these slopes were positively related to each other, whereas for other sets of components these slopes were negatively related to each other. These different contributions showed patterns across participants. For example, in readers for whom interest plays a relatively large role in how much they like a story, sadness will generally play a relatively weak role. This suggests that readers differ in what drives them to positively evaluate stories.

When we look at the individual variation in the associations between specific components and liking, we see that this association can be strong in some readers, but weak or even negative in other readers. This raises the question whether the assessed components of appreciation capture all reasons people like stories, or that there are other elements that also play into evaluations of stories. One likely possibility is that more cognitive (rather than affective) routes of aesthetic processing, such as foregrounding or stylistic elements in stories, contribute to the evaluation of literary story as well, and perhaps even more strongly in readers who respond weakly or negatively to the components assessed here.

Looking at the individual variation in the association between sadness and liking specifically, we found that readers differed in how negative emotions were related to their evaluations of stories. In some readers, negative emotions (sadness) in response to stories lead them to like those stories more, whereas for others negative emotions in response to stories lead to a decrease in liking. The association between negative emotions and liking is reminiscent of the phenomenon of *mixed emotions* in literary reading: It is possible to feel sadness (often experienced as a negative, unappreciated emotion), but perceive this as an enjoyable experience, for example in “bittersweet” situations (e.g., Larsen & McGraw, 2011; Oceja & Carrera, 2009; Schimmack, 2001). An example of mixed emotions in response to fiction can be found in the work by Hanich and colleagues (2014), which showed that in the context of film, experienced sadness (considered to be a negative emotion) is strongly positively correlated to enjoyment (a positive evaluation). The authors subsequently hypothesized that the correlation between sadness and enjoyment may not be a direct relationship, but may rather be mediated by the feeling of “being moved”. To elaborate, stories may elicit a feeling of sadness, which in turn contributes to the feeling of being moved, which is evaluated as a positive feeling. This way sadness can positively contribute to enjoyment, but *only* if this sadness results in or is interpreted as a feeling of being moved.

The paradoxical relationship between negative emotions and enjoyment is elaborated on by Menninghaus and colleagues (2017) in the *Distancing-Embracing model*. They state that the exceptional quality of art in being capable of leading to enjoyment through negative emotion lies in the processes of *distancing* and *embracing*. In this model, distancing refers to the sense of control art viewers feel when interacting with negatively valenced art: Viewers are aware that they can step away and stop looking as soon as they experience too many negative emotions due to the art work. This way they are confident they can distance themselves from these negative feelings before getting overwhelmed. Because of this process of distancing, art viewers can ultimately embrace an art work and the negative emotions associated with it. This might be through a feeling of being moved, or due to a process of cognitive dissonance resolution. A viewer may implicitly reason: This piece of art is eliciting negative emotions, and yet I am choosing to look at it, therefore I must like it. This way, in the aesthetic appreciation of art and literature, negative and positive emotions can both contribute to a positive evaluation of the object in question.

Indeed, as mentioned above, we found readers who displayed positive associations between (negative) emotions and liking, indicating that the processes of distancing and embracing when dealing with mixed emotions might influence “story liking” in *some* readers. However, there are also quite some readers who show a negative association between negative emotion in response to stories and liking, or are indifferent to negative emotion. The processes of distancing and embracing, and the phenomenon of mixed emotions therefore do not seem to manifest themselves equally in all readers.

Interestingly, individual variation in the relationships between the appreciation components and liking was not related to the experiential process of story world absorption (which conceptually differs from aesthetic experiences^6^) or to measures of daily life reading habits and print exposure. Although readers varied with respect to the relationship between aesthetic experiences and story liking, this did not translate to other measures often used in reading research (i.e., story world absorption, reading habits, print exposure). Apparently, aesthetic experiences are not directly associated with absorption, reading habits and print exposure, and they should not be used to make predictions about one another.

### Cognitive and Affective Routes to Aesthetic Appreciation

As elaborated on in the introduction, there are several theories and models of aesthetic appreciation that highlight the different routes to appreciation (Chatterjee & Vartanian, 2016; Jacobs, 2015a; Leder et al., 2004). Both affective (e.g., emotions elicited by a narrative) and cognitive (e.g., being intellectually challenged by a narrative) processing can contribute to the evaluation of a narrative. These different processing styles can interact in readers (or readers may prefer one style over the other), leading to different evaluations of the same narratives by different readers. In our study we find that, indeed, interaction between styles occurs in at least some readers, as both affective (e.g., sadness, amusement) and cognitive (e.g., interest) processes can be associated with general liking scores in one reader.

### Limitations and Directions for Future Research

It is important to note that the five components of appreciation measured in this paper, although a good start when it comes to measuring appreciation more comprehensively, will not be the *only* contributors to a reader’s eventual evaluation. Especially the cognitive elements of aesthetic appreciation (Chatterjee & Vartanian, 2016; Jacobs, 2015a; Leder et al., 2004) were not sufficiently captured in the adjectives derived from the study by Knoop and colleagues (2016) and may contribute to liking just as much as the components studied here (or perhaps even more strongly in readers who display low associations between the five components as measured in the current study and liking).

To address these limitations, it thus seems important that the cognitive processes involved in appreciation are investigated more thoroughly in future research. For example, the degree to which a story is experienced as intellectually challenging or stylistically striking is not captured in the adjectives used in the current study. In this context, the judgment of *beauty* should receive special attention. As we simply asked participants to rate the stories for being *beautiful*, without defining beauty as either stylistic or emotional beauty, we cannot tell what participants’ spontaneous criteria were when deciding on a rating for beauty (and thus whether this rating reflected a cognitive or emotional aesthetic process).

When studying individual variation in routes to appreciation, we can distinguish two sub-questions. In the current study, we have investigated *how* participants vary in their routes to liking. We have seen that aesthetic processes can be positively associated with liking in some participants, and negatively associated with liking in other participants. An open question with regard to the individual variation between readers as found in our analyses is *why* readers vary in their routes to liking. Leder and colleagues (2014) state that level of expertize is an important factor determining whether someone will prefer a cognitive processing style over an affective processing style. Therefore, we hypothesized that reading habits or print exposure would be associated with the individual variation between readers. However, in our results there is no indication that the differences between readers are due to their expertize, despite substantial variation in our sample. Both reading habits and print exposure could not sufficiently explain the differences between readers in the relationships between the components and liking.

Further exploration of the variation between readers could perhaps shine a light on different types of readers that may react differently to aesthetic experiences. For example, it would be interesting to answer the question whether there are mainly cognitively driven (i.e., distanced) or mainly affectively driven (i.e., identifying) readers, as well as readers who are somewhere in between (Riddell & van Dalen-Oskam, 2018). In a future experiment studying why participants differ in their routes to liking, it would be interesting to let participants read and rate a larger number of texts (perhaps also including texts of different genres). This would also address an important limitation of the current study: As the data in our study were not sampled across genres (and participants each read only 2–4 stories), we cannot generalize across genres. Therefore, no conclusions about genre differences could be made based on these data. A future study in which the stories are thoroughly sampled for genre differences would help shed light on any story or genre differences.

Pinpointing *how* and *why* readers vary in their routes to liking could in the future perhaps also help directing individuals to books or stories that they will like, through the use of recommender systems: for example, readers that enjoy sad stories (or who have characteristics that are associated with enjoying sad stories) could be recommended to read books liked by similar readers, whereas readers who prefer amusing stories would receive different recommendations (e.g., Faridani et al., 2017). This could result in more enjoyable reading experiences, which has been associated with a higher inclination to read again (Mol & Jolles, 2014), which in turn has been positively associated with school success (Chiu & McBride-Chang, 2006; Mol & Jolles, 2014; Retelsdorf et al., 2011) second language learning (Lao & Krashen, 2000; Lee et al., 2015; Yamashita, 2008), and social cognition and empathy (Fong et al., 2013; Johnson et al., 2013; Mar & Oatley, 2008; Oatley, 2016).

### Conclusions

Looking at the findings from this study, it is important to note that, while we do not contest the merit of any theoretical model of appreciation, there is a danger of “overfitting” these models to an “idealized reader.” We show that readers can have strikingly different reasons for indicating that they like a story or not. As a consequence, a simple question about the “liking” of a particular story will not inform us about the variation in the reading experiences that readers have. Our findings have illustrated how these individual differences contribute to evaluations, and have provided an example of how these differences could be quantitatively and empirically tested. This work might therefore motivate future empirical approaches to establishing individual differences in appreciation to get to a deeper understanding of what it means to “like” a story.

## Supplementary Information

**Additional file 1: Synopsis.** Synopsis of the nine stories used in the experiments

**Additional file 2: Fig. S1.** Individual (By-Participant) Posterior Distributions of the Relationship Between Interest and Liking. Note: Code for this figure is adapted from https://www.rensvandeschoot.com/tutorials/brms-started/.

**Additional file 3: Fig. S2.** Individual (By-Participant) Posterior Distributions of the Relationship Between Sadness and Liking. Note: Code for this figure is adapted from https://www.rensvandeschoot.com/tutorials/brms-started/.

**Additional file 4: Fig. S3.** Individual (By-Participant) Posterior Distributions of the Relationship Between Suspense and Liking. Note: Code for this figure is adapted from https://www.rensvandeschoot.com/tutorials/brms-started/.

**Additional file 5: Fig. S4.** Individual (By-Participant) Posterior Distributions of the Relationship Between Amusement and Liking. Note: Code for this figure is adapted from https://www.rensvandeschoot.com/tutorials/brms-started/.

**Additional file 6: Fig. S5.** Individual (By-Participant) Posterior Distributions of the Relationship Between Beauty and Liking. Note: Code for this figure is adapted from https://www.rensvandeschoot.com/tutorials/brms-started/.

## Data Availability

The datasets analyzed during the current study are available in the Open Science Framework, https://osf.io/h3ct6/.

## Abbreviations

NCPM: Neurocognitive poetics model
AESTHEMOS: Aesthetic emotions scale
SWAS: Story world absorption scale
ART: Author recognition test
PCA: Principal components analysis
KMO: Kaiser–Meyer–Olkin measure
IQR: Interquartile range
MAD: Median absolute deviation
CI: Credible interval

## Footnotes

1 The adjectives that we did not select, were either specific to different genres (theatre, poetry), or were not applicable to the stories that were used in our research (e.g., romantic, short, succinct).

2 Multilevel models are also known as mixed models or hierarchical models. The principle behind these types of models is that they are distinguishing a population level and a group level (in this case accounting for inter-participant and inter-story random effects).

3 All components were incorporated in the same model, to account for any shared variance between the components. Note, however, that this also means that all results of the components are after controlling for the other components, possibly reducing the effect sizes for the associations between all components and liking.

4 We report the median and credible interval for the posterior distribution of the standard deviation of the individual slopes (across each group). The standard deviation of the slopes of the components (either across participants or across stories) indicates whether there is reason to assume that the slopes vary between groups (i.e., participants, stories).

5 There were no effects on any of the subscales of the SWAS either.

6 Aesthetic appreciation and absorption are separated in all leading models of empirical aesthetics. One example that is particularly relevant for the present study is the Neurocognitive Poetics model by Jacobs (2015b). According to the Neurocognitive Poetics Model (NCPM; Jacobs, 2015b) aesthetic appreciation and narrative absorption are related to two different modes of literature processing. The point made by Jacobs, is that literary texts can contain elements that evoke either “fast” processing, via the so-called affective route, or “slow” processing, via a cognitive processing route. Fast processing can be evoked by elements such as suspenseful, emotional, and empathy-inducing passages in stories, and will lead to experiences such as narrative absorption in readers. Slow processing can be evoked by elements of foregrounding in stories, such as stylistic devices and defamiliarization. This processing mode will lead to aesthetic experiences, such as aesthetic appreciation of stories. Here, we have defined absorption as “an experiential state in which readers are focused on reading and the content of what is read. […] if the reading process feels effortless, readers experience a narrative world and feel for or with characters, and mental imagery is rich and vivid.” In this sense, absorption coincides with the fast route of the NCPM. Aesthetic appreciation, however, is not limited to the fast route of the NCPM, but contains both emotional (fast route) and cognitive or aesthetic (slow route) processes.
