# Supplementary material for: Different routes to liking: how readers arrive at narrative evaluations
Source: Cogn Res Princ Implic. 2022 Jul 30;7:72. doi: 10.1186/s41235-022-00419-0 (PMC9339064; doi:10.1186/s41235-022-00419-0)
Supplement: Supplementary file 1 — Additional file 1: Synopsis. Synopsis of the nine stories used in the experiments [file 41235_2022_419_MOESM1_ESM.docx]

**Appendix A: Synopsis of the nine stories used in the experiments**

The people that had everything delivered

The story recounts the experiences of a man who lives in an apartment building in Amsterdam. His neighbors rent out their apartment while they are on holiday for the Christmas days, and a morbidly obese British couple stays there. When the wife has a heart attack she has to be lifted out of the apartment by a firetruck, as there is no elevator in the building.

The Chinese wedding

In this story, a Dutch male student is best man at his roommate’s wedding to a Chinese exchange student. He ends up spending the day with the parents of the bride who don’t speak a word of English, which leads to some very awkward moments.

Symbols and signs

This story tells about an older Russian Couple, that lives in the USA. Their son is hospitalized, and on his birthday, they can’t visit him as he just attempted suicide. The rest of the day, the mother is reminiscing about her son’s life, and later that night the father decides that he wants to bring him home to take care of him by themselves.

It is mouse

In this story, a preschool teacher is very annoyed by one of her students which results in her trying to ignore the child. This results in the child dying in an unhappy accident at the preschool, due to the teacher’s negligence.

How the wolves dance

This story is about someone who wakes up during the night to discover dancing wolves in his living room.

The substitute

In this story, a girl is sitting in a cafe, and she thinks that she is being stalked by a man who eventually turns out to be an acquaintance – as he used to be her substitute teacher. The story ends on the cliffhanger that perhaps, after all, he is not exactly who he claims he is.

She is everywhere

This story is about a man in the library in Utrecht, who has the feeling that he is being followed around by his ex (the subtext is that he has a psychosis, since he is not really being followed around).

Moped on sea

This is a surrealistic story about a boy on a boat and his encounter with a man riding a moped at sea in the middle of the night.

God and the judge of the insane

In this story, the author narrates the story of a mentally instable man who is convinced that he is God, and believes that therefore all his excrements are holy and should not be thrown away. Apart from that, he terrorizes the neighborhood, leading to his institutionalization later on in the story, after which he finally seems to realize that he was mistaken in thinking that he was God.

**Appendix B**

[Insert Figure Appendix B Figure 1 here]

**Figure B1**

*Individual (By-Participant) Posterior Distributions of the Relationship Between Interest and Liking*

Note: Code for this figure is adapted from https://www.rensvandeschoot.com/tutorials/brms-started/.

[Insert Figure Appendix B Figure 2 here]

**Figure B2**

*Individual (By-Participant) Posterior Distributions of the Relationship Between Sadness and Liking*

Note: Code for this figure is adapted from https://www.rensvandeschoot.com/tutorials/brms-started/.

[Insert Figure Appendix B Figure 3 here]

**Figure B3**

*Individual (By-Participant) Posterior Distributions of the Relationship Between Suspense and Liking*

Note: Code for this figure is adapted from https://www.rensvandeschoot.com/tutorials/brms-started/.

[Insert Figure Appendix B Figure 4 here]

**Figure B4**

*Individual (By-Participant) Posterior Distributions of the Relationship Between Amusement and Liking*

Note: Code for this figure is adapted from https://www.rensvandeschoot.com/tutorials/brms-started/.

[Insert Figure Appendix B Figure 5 here]

**Figure B5**

*Individual (By-Participant) Posterior Distributions of the Relationship Between Beauty and Liking*

Note: Code for this figure is adapted from https://www.rensvandeschoot.com/tutorials/brms-started/.
